# Association of early-term birth and breastfeeding practices with nutritional outcomes in singleton term infants: a multicenter cross-sectional study

**DOI:** 10.1186/s13006-024-00653-w

**Published:** 2024-07-02

**Authors:** Li Zhang, Hui-Juan Liu, Ping Li, Yi Liu, Ting Zhang, Jin-Yi Zhu, Hong-Mei Zhu, Ya-Ping Zhou, Hai-Jun Wang, Yan Li

**Affiliations:** 1 Research Center for Child Health, Department of Child Health Care, Key Laboratory of Birth Regulation and Control Technology of National Health Commission of China, Shandong Provincial Maternal and Child Health Care Hospital Affiliated to Qingdao University, No. 12675, Jingshi Road, Jinan, Shandong 250014 China; 2 Department of Maternal and Child Health, School of Public Health, National Health Commission Key Laboratory of Reproductive Health, Peking University, Beijing, China; 3 School of Clinical Medicine, Affiliated Hospital of Weifang Medical University, Weifang Medical University, Weifang, China; 4 Department of Child Health Care, Tengzhou Maternal and Child Health Care Hospital, Tengzhou, China

**Keywords:** Gestational age, Early-term, Breastfeeding, Malnutrition, Obesity

## Abstract

**Background:**

Limited research has explored the associations of gestational age (GA) and breastfeeding practices with growth and nutrition in term infants.

**Methods:**

This multicenter cross-sectional study recruited 7299 singleton term infants from well-child visits in Shandong, China, between March 2021 and November 2022. Data on GA, gender, ethnicity, birth weight, parental heights, gestational diabetes and hypertension, age at visit, breastfeeding practices (point-in-time data at visit for infants < 6 months and retrospective data at 6 months for infants ≥ 6 months), complementary foods introduction, infant length and weight, were collected. 7270 infants were included in the analysis after excluding outliers with Z-scores of length (LAZ), weight or weight for length (WLZ) <-4 or > 4. Linear regression models adjused for covariates explored the impact of GA and breastfeeding practices on LAZ and WLZ, while logistic regression models evaluated their effect on the likelihood of moderate and severe stunting (MSS, LAZ<-2), moderate and severe acute malnutrition (MSAM, WLZ<-2) and overweight/obesity (WLZ > 2). Sensitivity analysis was conducted on normal birth weight infants (2.5–4.0 kg).

**Results:**

Infants born early-term and exclusively breastfed accounted for 31.1% and 66.4% of the sample, respectively. Early-term birth related to higher WLZ (< 6 months: *β* = 0.23, 95% confidence interval (CI): 0.16, 0.29; ≥6 months: *β* = 0.12, 95% CI: 0.04, 0.20) and an increased risk of overweight/obesity throughout infancy (< 6 months: OR: 1.41, 95% CI 1.08, 1.84; ≥6 months: OR: 1.35, 95% CI 1.03, 1.79). Before 6 months, early-term birth correlated with lower LAZ (*β*=-0.16, 95% CI: -0.21, -0.11) and an increased risk of MSS (OR: 1.01, 95%CI 1.00, 1.02); Compared to exclusive breastfeeding, exclusive formula-feeding and mixed feeding linked to lower WLZ (*β*=-0.15, 95%CI -0.30, 0.00 and *β*=-0.12, 95%CI -0.19, -0.05, respectively) and increased risks of MSAM (OR: 5.57, 95%CI 1.95, 15.88 and OR: 3.19, 95%CI 1.64, 6.19, respectively). Sensitivity analyses confirmed these findings.

**Conclusions:**

The findings emphasize the health risks of early-term birth and the protective effect of exclusive breastfeeding in singleton term infants, underscoring the avoidance of nonmedically indicated delivery before 39 weeks and promoting exclusive breastfeeding before 6 months.

## Background

The “First 1000 Days” and “Developmental Origins of Health and Disease” theories emphasize the critical role of early-life nutrition and growth in shaping long-term health outcomes [1, 2]. Therefore, investigating the factors influencing child growth to identify early targets for malnutrition prevention and intervention, and promote optimal growth in early life, has long been a focus for pediatricians and society. While genetic and environmental factors are known to be essential in shaping growth trajectories [3], there is currently no definitive conclusion on how these factors specifically influence child growth. In particular, considering the stagewise nature of child growth, exposure and susceptibility to risk factors might vary at different stages. For example, the effects of introducting complementary foods usually emerge after 6 months of age [4], while some perinatal factors, such as maternal nutrition and illness, as well as neonatal complications, could tend to affect or have a greater impact on younger infants. At present, specific risk factors influencing infant growth stratified by age are yet to be elucidated. In addition, previous studies have focused primarily on the factors influencing the growth of high-risk infants, particularly preterm and low birth weight infants [5, 6]. There are limited studies that have explored the factors affecting early-life growth in term infants, as they are typically considered “healthy” from birth [7]. However, given that term infants account for the vast majority of the population (90%) [8], investigating the factors associated with their growth could make a significant contribution to the overall health status of the population.

A substantial body of research on preterm infants has established a clear link between lower gestational age (GA) at birth and elevated risks of neonatal morbidity and mortality, as well as detrimental effects on both short-term and long-term physical growth and neurological development [9, 10]. Moreover, over the past decade, the impact of GA on the health of term infants has gained increasing attention. Given the heightened risk of neonatal complications and hospitalization for infants born at less than 39 weeks of gestation, the American College of Obstetricians and Gynecologists introduced the “39-week rule” in 2009 to reduce nonmedically indicated elective cesarean delivery before 39 weeks [11]. Additionally, growing evidence suggests that, similar to late preterm infants (GA 34–36 weeks), early-term births (GA 37–38 weeks) also exhibit impairment in long-term neurological outcomes [12, 13]. These findings reinforced the recommendation to delay delivery until 39 weeks. However, the existing evidence primarily focuses on the association between early-term birth and perinatal and long-term neurological risks, with limited literature on the correlation between early-term birth and physical growth and nutritional status in children. There is currently scarce research on whether early-term birth is correlated with adverse physical growth and an increased risk of malnutrition.

Early life nutrition has always been considered the most important regulable environmental factor affecting the growth of infants [14]. However, given the heterogeneity in research related to nutrition and its impact on growth outcomes [15] and the complex and intertwined relationships between different feeding practices (such as breastfeeding and complementary feeding) [16], there is currently no clear conclusion regarding the influence of early-life feeding practices on the risk of malnutrition and physical growth at different stages of infancy.

Therefore, this study aims to explore the associations of GA at birth (early-term: 37–38 weeks, and full-term: GA 39–41 weeks) and feeding pattern (exclusive breastfeeding, mixed feeding and exclusive formula-feeding) with anthropometric indicators and the risk of malnutrition in singleton term infants at two stages of infancy (0–5 months and 6–12 months) while controlling for potential influencing factors.

## Methods

### Study design

This was a multicenter cross-sectional study. The data utilized in this study, including information on breastfeeding practices, birth characteristics, physical and nutritional indicators, etc., were collected through the survey of “Infant Body Composition Study” [17]. These research findings are consistent with the broader objectives of the “Infant Body Composition Study”.

### Participants

All participants were recruited from well-child health visits at four medical institutions between March 2021 and November 2022: Jinan, Liaocheng, Tengzhou, and Dongying in Shandong Province, China. Healthy singleton term infants who were born between 37^+ 0^ and 41^+ 6^ weeks of GA, and whose age at the time of the visit ranged from 0 to 12 months, were recruited. Exclusion criteria included congenital malformations, syndromes, and short- or long-term diseases. In our local context, infant well-child visits are generally scheduled at 42 days, 3 months, 6 months, 8 or 9 months, and 12 months post-birth, leading to a concentration of participants within these age intervals.

Ethical approval was granted from the Medical Ethics Committee of The First Affiliated Hospital of Shandong First Medical University (YXLL-KY-2022 (017)) and the Medical Ethics Committee of Shandong Provincial Maternal and Child Health Care Hospital Affiliated with Qingdao University (No. 2023-025). Written informed consent was obtained from all participants of their parents.

### Nutrition practices

Mothers of the participants received nutritional and feeding guidance based on current World Health Organization (WHO) guidelines from pediatricians at routine well-child visits [18]. The recommendations included: (1) exclusively breastfeeding for the first 6 months of life and (2) the introduction of complementary foods at 6 months, along with continued breastfeeding up to 2 years of age or beyond. This study obtained information on the implementation of feeding patterns before 6 months of age and the timing of complementary foods introduction through a questionnaire survey during the visit.

### Data collection

Information about infants at birth included ethnicity (“Han”, others), gender (“female”, “male”), birth weight (kg), and GA (week). This information was obtained from the medical records of child health care centers or birth certificates of infants. In this study, we reclassified GA into two categories for analysis: 0. “full-term”, defined as GA 39^+ 0^–41^+ 6^ weeks, and 1. “early-term”, defined as GA 37^+ 0^–38^+ 6^ weeks [11].

Data on nutritional practices was obtained from a parental questionaire at visit, which included breastfeeding practices and complementary foods introduction. There were five categories for feeding patterns in the questionnaire: “1. exclusive breastfeeding”, “2. mainly breastfeeding”, “3. breastfeeding accounts for half”, “4. mainly formula-feeding”, and “5. exclusive formula-feeding”, which were reclassified as “1. exclusive breastfeeding”, “2. mixed feeding” (including “mainly breastfeeding”, “half breastfeeding”, and “mainly formula-feeding” in the questionnaire), and “3. exclusive formula-feeding”) for analysis. For infants under 6 months old, parents were requested to document the current breastfeeding routines (point-in-time data), i.e., the breastfeeding practices in previous 24 h before the visit; while for infants 6 months and older, retrospective data about the breastfeeding practices when they got 6 months age was documented. Complementary foods introduction was documented as “no” or “yes” for infants under 6 months old and as “introduced at 4–5 months” or “introduced at ≥ 6 months” for infants 6 months and older.

Maternal information during gestation included gestational diabetes mellitus (GDM, “no”, “yes”) and gestational hypertension (“no”, “yes”), which were also documented through the parental questionnaire at the visit.

All participants have complete birth information documented in their medical records or birth certificates. Parental questionnaires were administered on-site by trained staff during recruitment and thoroughly checked for relevant variables to guarantee the completeness of data for all participants.

### Anthropometric measurements

The measurements of length and weight for all infants were conducted according to standard procedures, as detailed in our previous study [17]. The Z-scores of length, weight and weight for length were calculated in accordance with WHO growth standards and recorded as LAZ, WAZ and WLZ, respectively. Three types of malnutrition were documented: moderate and severe stunting (MSS) [19], defined as LAZ <-2; moderate and severe acute malnutrition (MSAM) [20], defined as WLZ <-2; and overweight and obesity, defined as WLZ > 2.

### Data sampling

**Fig. 1:**
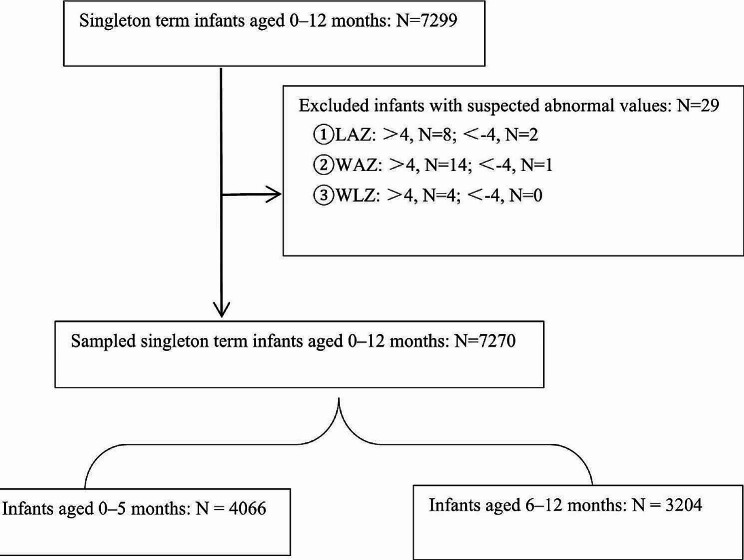
Flowchart of data sampling

A total of 7299 singleton term infants with complete data were enrolled in the study. To ensure data quality and accuracy, rigorous data cleaning was conducted: any LAZ, WAZ, or WLZ values outside the range of -4 to 4 were considered potentially suspicious outliers. Participants displaying one or more of these outliers were excluded from the final analysis, resulting in the removal of 29 participants. Among the remaining 7270 infants, 4066 were aged 0–5 months and 3204 were aged 6–12 months (Fig. 1).

### Statistical analyses

Categorical variables are presented as numbers (n) and percentages (%), while continuous variables are presented as the means (standard deviations) or means (95% CIs).

In both age groups, covariates adjusted for included gender, ethnicity, birth weight, GDM, gestational hypertension, parental heights, and age at the visit. Considering that most infants under 6 months did not reach the recommended time for introducing complementary foods, the covariate complementary foods introduction was only included in the analysis of infants aged ≥ 6 months. Linear regression models were utilized to investigate the correlation of GA at birth (full-term and early-term) and feeding practices (exclusive breastfeeding, mixed feeding and exlusive formula-feeding) with LAZ and WLZ, with β values indicating these relationships. Logistic regression models were employed to explore the associations of GA at birth and feeding practices with the risks of MSS, MSAM, and overweight/obesity, with OR values representing the likelihood of these outcomes. A significance level of *P* < 0.05 was considered to indicate statistical significance.

Given the potential influence of macrosomic infants (birth weight > 4.0 kg) and low birth weight infants (birth weight < 2.5 kg) on observed outcomes, we performed a sensitivity analysis in children of normal birth weight (2.5–4.0 kg) to assesse the robustness of study findings. The covariates adjusted for and the statistical methods utilized in the sensitivity analysis were entirely consistent with those previously described, with the exception of incorporating children with normal birth weight.

All the statistical analyses were conducted using IBM SPSS Statistics 21 software (Chicago, IL, USA) and R software (version 4.0.3; creator: John Chambers and colleagues; location: Jersey City, NJ, USA).

## Results

### General characteristics, growth and nutritional outcomes

The general characteristics, growth and nutritional outcomes of 4066 infants aged 0–5 months and 3204 infants aged 6–12 months are shown in Table 1. The proportion of early-term infants in the sample was 31%. Exclusive breastfeeding was the predominant feeding pattern in both the < 6 months and ≥ 6 months age groups, constituting approximately 66% of the total sample. Among infants aged ≥ 6 months, 82.0% had complementary foods introduced at or after 6 months of age. The LAZ, WAZ, and WLZ for both groups were all above zero, indicating that the growth levels of this population surpassed the WHO standards. The prevalence of MSS and MSAM was very low in both groups, at approximately 1%; however, the rates of overweight and obesity were relatively high, with 7.0% and 8.7% for infants aged 0–5 months and 6–12 months, respectively.

**Table 1:** General characteristics, growth and nutritional outcomes of 7270 healthy singleton term infants in China

	0–5 months	6–12 months
N	4066	3204
Gender (boy)	2158 (53.1%)	1664 (51.9%)
Ethnicity (Han)	4043 (99.4%)	3197 (99.8%)
Age at visit (month)	2.63 (1.59)	8.96 (2.42)
Gestational age (GA, week)	39.03 (1.09)	39.11 (1.11)
Categorized by GA		
Early-term (GA, 37–38 wks)	1304 (32.1%)	957 (29.9%)
Full-term (GA, 39–41 wks)	2762 (67.9%)	2247 (70.1%)
Birth weight (kg)	3.33 (0.42)	3.34 (0.42)
Low birth weight (< 2.5 kg)	79 (1.9%)	49 (1.5%)
Normal birth weight (2.5–4.0 kg)	3798 (93.4%)	2987 (93.2%)
Macrosomia (> 4.0 kg)	189 (4.6%)	168 (5.2%)
Feeding patterns within 6 months^※^		
Exclusive breastfeeding	2699 (66.4%)	2125 (66.3%)
Mixed feeding	1182 (29.1%)	869 (27.1%)
Exclusive formula feeding	185 (4.5%)	210 (6.6%)
Timing of complementary food introduction		
Not yet	3935 (96.8%)	-
4–5 months	131 (3.2%)	577 (18.0%)
≥ 6 months	-	2627 (82.0%)
Gestational diabetes mellitus	522 (12.8%)	235 (7.3%)
Gestational hypertension	112 (2.8%)	87 (2.7%)
Father height (cm)	175.20 (5.11)	175.63 (5.08)
Mother height (cm)	162.12 (5.05)	162.38 (4.95)
Length (cm)	59.66 (4.90)	71.83 (3.92)
Weight (kg)	6.06 (1.50)	9.14 (1.27)
LAZ	0.30 (0.95)	0.42 (1.01)
WAZ	0.48 (0.94)	0.58 (1.03)
WLZ	0.43 (1.03)	0.54 (1.05)
MSS (LAZ < -2)	41 (1.0%)	29 (0.9%)
MSAM (WLZ < -2)	42 (1.0%)	21 (0.7%)
Overweight and obesity (WLZ > 2)	283 (7.0%)	278 (8.7%)

*Abbreviations* GA, gestational age; LAZ, length-for-age Z-score; MSAM, moderate and severe acute malnutrition; MSS, moderate and severe stunting; WAZ, weight-for-age Z-score; WLZ, weight-for-length Z-score. LAZ, WAZ and WLZ were calculated according to the WHO growth standards

※For infants < 6 months, the breastfeeding practices in previous 24 h before the visit (point-in-time data) were documented; for infants ≥ 6 months, breastfeeding practices at 6 month of age (retrospective data) was documented

### Associations of GA and feeding patterns with anthropometric indicators and malnutrition

After adjusting for covariates, the associations of GA and feeding patterns with anthropometric indicators and malnutrition are shown in Table 2.

For infants aged < 6 months, early-term birth was negatively correlated with LAZ (*β*=-0.16, 95% CI -0.21, -0.11), positively correlated with WLZ (*β* = 0.23, 95% CI 0.16, 0.29), and was associated with greater risks of MSS (OR:1.01, 95%CI 1.00, 1.02) and overweight/obesity (OR: 1.41, 95% CI 1.08, 1.84). Compared to exclusive breastfeeding, mixed feeding and exclusive formula-feeding were both negatively correlated with WLZ (*β*=-0.12, 95% CI -0.19, -0.05 for mixed feeding; *β*=-0.15, 95%CI -0.30, 0.00 for exclusive formula-feeding) and were linked to increased risks of MSAM (OR: 5.57, 95% CI 1.95, 15.88 for exclusive formula-feeding; OR: 3.19, 95% CI 1.64, 6.19 for mixed feeding).

For infants aged ≥ 6 months, early-term birth exhibited a positive correlation with WLZ (*β* = 0.12, 95% CI 0.04, 0.20) and a greater risk of overweight/obesity (OR: 1.35 95% CI 1.03, 1.79), while not showing significant correlation with LAZ or the risk of MSS. However, except that mixed feeding showed a positive correlation with LAZ (*β* = 0.10, 95% CI 0.03, 0.17), the associations between feeding practices and the risks of MSAM were not significant during this period.

There were no statistically significant differences in the effects of mixed feeding and exclusive formula-feeding on all outcome measures during infancy.

**Table 2:** Association of gestational age and feeding practices with growth and nutritional outcomes in 7270 singleton term infants in China

	Variables	LAZ	WLZ	MSS (LAZ<-2)	Overweight/obesity (WLZ > 2)	MSAM (WLZ<-2)
		β (95%CI)	β (95%CI)	OR (95%CI)	OR (95%CI)	OR (95%CI)
**< 6 months** (***n***=4066)^**※**^	**Gestational age**					
	Full-term	Ref	Ref	Ref	Ref	Ref
	Early-term	-0.16 (-0.21, -0.11) ^***^	0.23 (0.16, 0.29) ^***^	1.01 (1.00, 1.02) ^*^	1.41 (1.08, 1.84) ^*^	0.90 (0.46, 1.76)
	**Feeding pattern** ^**#**^					
	Exclusive breastfeeding	Ref	Ref	Ref	Ref	Ref
	Mixed feeding	0.01 (-0.04, 0.06)	-0.12 (-0.19, -0.05) ^**^	1.01 (1.00, 1.01)	0.89 (0.67, 1.17)	3.19 (1.64, 6.19) ^**^
	Exclusive formula-feeding	0.09 (-0.02, 0.20)	-0.15 (-0.30, 0.00) ^*^	0.99 (0.98, 1.01)	0.86 (0.49, 1.52)	5.57 (1.95, 15.88) ^**^
**≥ 6 months (n=3204)** ^※^	**Gestational age**					
	Full-term	Ref	Ref	Ref	Ref	Ref
	Early-term	-0.01 (-0.08, 0.06)	0.12 (0.04, 0.20) ^**^	1.00 (0.99, 1.00)	1.35 (1.03, 1.79) ^*^	0.91 (0.35, 2.37)
	**Feeding pattern** ^**#**^					
	Exclusive breastfeeding	Ref	Ref	Ref	Ref	Ref
	Mixed feeding	0.10 (0.03, 0.17) ^**^	-0.03 (-0.11, 0.05)	1.00 (0.99, 1.01)	0.80 (0.60, 1.08)	0.64 (0.21, 1.95)
	Exclusive formula-feeding	0.07 (-0.05, 0.19)	-0.12 (-0.26, 0.03)	1.00 (0.98, 1.01)	0.60 (0.33, 1.11)	1.49 (0.33, 6.68)

*Abbreviations* LAZ, length-for-age Z-score; MSAM, moderate and severe acute malnutrition; MSS, moderate and severe stunting; WLZ, weight-for-length Z-score; CI, confidence interval; OR, odds ratio; Ref, Reference category

※The models for infants aged < 6 months were adjusted for gender, birth weight, age at visit, ethnicity, gestational hypertension, gestational diabetes mellitus, and parental heights. For infants aged ≥ 6 months, the models were further adjusted for the timing of complementary food introduction in addition to the aforementioned covariates

Level of signifcance: *P < 0.05, **P < 0.01, ***P < 0.001

#No statistical differences were observed in the effects of mixed feeding and exclusive formula-feeding on any of the outcomes

### Sensitivity analysis

The sensitivity analysis was performed on 6785 (93.3%) normal birth weight infants (2.5–4.0 kg). The results are presented in Table 3.

In infants with normal birth weights aged < 6 months, early full-term birth was associated with lower LAZ (*β*=-0.14, 95% CI -0.19, -0.09), higher WLZ (*β* = 0.23, 95% CI 0.16, 0.30), and increased risks of MSS (OR: 1.01, 95%CI 1.00, 1.01) and overweight/obesity (OR: 1.38, 95% CI 1.05, 1.82). Compared with exclusive breastfeeding, both exclusive formula-feeding and mixed feeding showed negative associations with WLZ (*β*=-0.18, 95% CI -0.34, -0.03 for exclusive formula-feeding; *β*=-0.13, 95% CI -0.20, -0.05 for mixed feeding) and higher risks of MSAM (OR: 6.48, 95% CI 2.20, 19.09 for exclusive formula-feeding; OR: 3.73, 95% CI 1.81, 7.67 for mixed feeding).

In infants with normal birth weights aged ≥ 6 months, early-term birth was linked to higher WLZ (*β* = 0.13, 95% CI 0.05, 0.21) and an increased risk of overweight/obesity (OR: 1.38, 95% CI 1.04, 1.83). Nevertheless, apart from mixed feeding demonstrating a positive correlation with LAZ (*β* = 0.09, 95% CI 0.02, 0.16) when compared to exclusive breastfeeding, there were no notable associations between feeding practices and outcomes in this timeframe.

There were no statistically significant differences in the effects of mixed feeding and exclusive formula-feeding on all outcome measures during infancy.

**Table 3:** Association of gestational age and feeding practices with growth and nutritional outcomes in 6785 singleton term infants with normal birth weight (2.5–4.0 kg) in China

	Variables	LAZ	WLZ	MSS (LAZ<-2)	Overweight/obesity (WLZ > 2)	MSAM (WLZ<-2)
		β (95%CI)	β (95%CI)	OR (95%CI)	OR (95%CI)	OR (95%CI)
**< 6 months** (***n*** = 3798) ^※^	**Gestational age**					
	Full-term	Ref	Ref	Ref	Ref	Ref
	Early-term	-0.14 (-0.19, -0.09) ^***^	0.23 (0.16, 0.30) ^***^	1.01 (1.00, 1.01) ^*^	1.38 (1.05, 1.82) ^*^	0.92 (0.45, 1.86)
	**Feeding pattern** ^**#**^					
	Exclusive breastfeeding	Ref	Ref	Ref	Ref	Ref
	Mixed feeding	0.02 (-0.04, 0.07)	-0.13 (-0.20, -0.05) ^**^	1.01 (1.00, 1.01)	0.88 (0.66, 1.18)	3.73 (1.81, 7.67) ^***^
	Exclusive formula-feeding	0.08 (-0.03, 0.19)	-0.18 (-0.34, -0.03) ^*^	0.99 (0.98, 1.01)	0.73 (0.39, 1.36)	6.48 (2.20, 19.09) ^**^
**≥ 6 months** (***n* = 2987)**^※^	**Gestational age**					
	Full-term	Ref	Ref	Ref	Ref	Ref
	Early-term	0.00 (-0.07, 0.07)	0.13 (0.05, 0.21) ^**^	1.00 (0.99, 1.00)	1.38 (1.04, 1.83) ^*^	0.94 (0.37, 2.41)
	**Feeding pattern** ^**#**^					
	Exclusive breastfeeding	Ref	Ref	Ref	Ref	Ref
	Mixed feeding	0.09 (0.02, 0.16) ^*^	0.00 (-0.08, 0.09)	1.00 (0.99, 1.01)	0.86 (0.63, 1.16)	0.65 (0.21, 1.99)
	Exclusive formula-feeding	0.09 (-0.04, 0.22)	-0.10 (-0.26, 0.05)	1.00 (0.98, 1.01)	0.62 (0.33, 1.17)	1.49 (0.33, 6.73)

*Abbreviations* LAZ, length-for-age Z-score; MSAM, moderate and severe acute malnutrition; MSS, moderate and severe stunting; WLZ, weight-for-length Z-score; CI, confidence interval; OR, odds ratio; Ref, Reference category

※The models for infants aged < 6 months were adjusted for gender, birth weight, age at visit, ethnicity, gestational hypertension, gestational diabetes mellitus, and parental heights. For infants aged ≥ 6 months, the models were further adjusted for the timing of complementary food introduction in addition to the aforementioned covariates

Level of signifcance: *P < 0.05, **P < 0.01, ***P < 0.001

#No statistical differences were observed in the effects of mixed feeding and exclusive formula-feeding on any of the outcomes

## Discussion

This multicenter observational study explored the associations of early-term birth and breastfeeding practices with growth and nutrition in singleton term infants aged < 6 months and ≥ 6 months, respectively, taking into account various potential risk factors that could impact infant growth, including parental heights, maternal health status, birth weight, gender, ethnicity and age at follow-up, etc. The stratification of age groups was considering the differences in growth velocity, potential risk factors, introduction of complementary foods, and the breastfeeding practices in infants before and after 6 months of age. For infants aged ≥ 6 months, the timing of complementary foods introduction was taken into account. These factors were included as covariates in the models to ensure that the conclusions drawn about the association between early-term birth and breastfeeding practices with infant growth and nutrition were more precise and dependable. For instance, pregnancy-related risk factors, such as gestational hypertension and GDM, have long been a concern for the health of fetuses and infants [21, 22]. Birth weight reflects the growth status and outcome of the fetal period, and its impact on short- and long-term health has been confirmed by numerous studies [7, 23] Considering the significant variations in growth rates, patterns, and potential nutritional risks among infants with different birth weights, this study not only considered birth weight as a critical covariate but also conducted sensitivity analyses within normal birth weight infants, ensuring more robust and reliable research findings. The timing of complementary foods introduction is considered important, as inappropriate nutritional intake can change infant growth rates, which have been identified as important risk factors for subsequent obesity [24] Therefore, we also included the timing of complementary foods introduction as one of the models’ covariates in infants aged ≥ 6 months, controlling for its potential effects.

After adjusting for the above-mentioned covariables, this study demonstrated a significant and independent association between early-term birth and overweight and obesity in singleton term infants throughout infancy. We found a 41% increase in the risk of overweight and obesity in early-term infants aged < 6 months and a 35% increase in those aged ≥ 6 months. Additionally, this study revealed an association between exclusive breastfeeding and a lower incidence of moderate and severe acute malnutrition in infants under 6 months of age. Specifically, compared to infants exclusively breastfed, infants fed with partial or exclusive formula faced 3.73 to 6.48 times the risk of moderate and severe acute malnutrition. These significant associations remained stable in the sensitivity analyses.

This evidence of an association between early-term birth and adverse health outcomes in terms of growth and nutrition is intriguing. As we know, the evidence supporting the decision to advocate for deliveries at 39 weeks or later primarily arises from (1) the correlation between early-term births and increased risks of neonatal morbidity, mortality, hospitalization, and extended hospital stays [11, 25] and (2) impaired long-term neurological development in early-term infants compared to full-term infants [26]. However, this evidence lacks confirmation of the association between early-term birth and adverse outcomes in children’s growth and nutrition. Nevertheless, a recent population study established a link between early-term birth and overweight and obesity in children hospitalized for endocrine and metabolic diseases [27]. However, to the best of our knowledge, there is limited evidence regarding the link between early-term birth and the risk of overweight and obesity in healthy singleton term infants. Our findings suggest that in the healthy term-born population, infants born before 39 weeks GA have an elevated risk of overweight and obesity as early as early infancy. Considering that (1) the steadily increasing prevalence of obesity has now become a significant burden for human health [28], (2) obesity is widely regarded as a multifaceted disease and the main risk condition for developing metabolic syndrome in both children and adults [29] and (3) in addition to excess nutritional intake and a sedentary lifestyle, other potential causes and mechanisms leading to obesity have not yet been fully elucidated [30]. The finding of the association between early-term delivery and a greater risk of overweight and obesity in infants may have important implications for the early intervention of overweight and obesity. However, the underlying mechanisms of the association between early-term birth and the risk of overweight and obesity remain unclear and require further exploration. Previous studies reported a correlation between smaller gestational age and slower length growth in late preterm infants (GA 34–36 weeks) [5, 31], which aligns with the association of early-term birth with poorer length growth in early-term infants observed in this study. Specifically, the significantly lower length growth accompanied by relatively normal weight growth in early-term infants than in full-term infants might be a direct cause of the increased risk of overweight and obesity in early-term infants. Even so, the potential mechanisms underlying the association between early-term birth and adverse linear growth and overweight and obesity remain unclear, necessitating further clinical and laboratory research. Overall, this study contributes to the growing body of evidence on the potential health risks of early-term delivery for offspring [12, 13], indicating that nonmedically indicated elective cesarean delivery before 39 weeks should be avoided.

MSAM (wasting) among infants under six months remains a major global health concern. Recent analyses of data from Demographic Health Survey in 56 countries estimates that 21.3% of infants under six months are wasted [32]. These infants are at increased risk of mortality, morbidity and poor growth and development in both the short and long term. Early-life nutrition plays a pivotal role in influencing the growth and development of infants [33] and is one of the most significant modifiable environmental factors [4]. Among these factors, breastfeeding has been convinced to be the most important intervention for improving malnutrition in infants and young children [34]. In addition, exclusive breastfeeding is a crucial public health intervention that not only provides optimal nutrition but also provides economic, social, and health benefits [35, 36]. However, evidence on the effect of breastfeeding promotion on growth and nutrition is equivocal [37]. This might be attributed to the ambiguity in defining breastfeeding practices (exclusive breastfeeding, predominant breastfeeding, and any/partial breastfeeding, etc.), as well as differences in age, ethnicity, and economic environments (low-, middle-, and high-income settings). However, the duration, intensity, and quantity and exclusivity of breastfeeding have been shown to be important in quantifying the benefits of breastfeeding for both children and mothers [38]. Our study suggested that compared to exclusive formula-feeding and mixed feeding, exclusive breastfeeding was linked to higher WLZ and the lowest risk of moderate and severe acute malnutrition in infants under 6 months old, without an increased risk of overweight and obesity. These findings emphasize the importance of the exclusivity of breastfeeding in infants under 6 months of age, highlighting the continued support and promotion of exclusive breastfeeding in this age group [35]. Similar to our findings, Rebhan et al. also demonstrated the lower WLZ in infants who were not breastfed compared to infants who were breastfed before 6 months of age [16]. However, a systematic review and meta-analysis indicated that breastfeeding interventions led to a slight but significant reduction in body mass index/Weight-for-Height Z-scores (Z-score mean difference: -0.06, 95%CI: -0.12, 0.00)) in children under 5 years [39]. Nonetheless, this effect was observed only in low- and high-income countries, but not in middle-income countries. Additionally, this effect was not observed in infants under 6 months of age. Considering that: (1) our study was an observational study, not an intervention study promoting breastfeeding; (2) our study was conducted in China, a middle-income country, rather than low- and high-income countries; (3) our study included infants aged 0–12 months, rather than children under 5 years old; these factors may contribute to the discrepancies in our findings compared to the aforementioned meta-analyses [39]. Nonetheless, the findings of our study provide additional evidence supporting the beneficial effects of exclusive breastfeeding during the first six months of life [34, 35]. However, the specific mechanisms for this correlation are yet to be elucidated. Breastfeeding might promote infant growth and nutrition through its nutritional properties and by reducing incidence and severity of potentially growth-affecting infections, especially diarrhoea and respiratory diseases [36]. Moving forward, further investigation is crucial to explore the evidence and potential mechanisms behind the nutritional and growth benefits of exclusive breastfeeding in infants under 6 months old in China and similar middle-income countries.

The strengths of this study lie in several key aspects. First, the research was carried out using a substantial sample of singleton term infants from medical centers located in four cities in northern China, with the demographic characteristics of the sample comparable to those of a national study [40]. Moreover, the feeding and nutritional practices were in line with international recommendations [18]. As a result, the data obtained from this study can be considered representative of healthy singleton term infants without significant growth restrictions in China and in countries with similar economic and cultural backgrounds. Second, this study accounted for important influencing factors that could impact infant growth at various stages, integrating them as covariates in the models. Furthermore, sensitivity analysis was performed to ensure the robustness and reliability of the research findings. Third, the evidence from this study is compelling and enhances our comprehension of the health risks associated with early-term infants and the benefits of exclusive breastfeeding, some of which were previously overlooked. This includes the link between early-term birth and a heightened risk of overweight and obesity, emphasizing the necessity for targeted interventions to prevent nonmedically indicated early-term deliveries.

The present study also has several limitations that should be considered when interpreting the findings. First, the observational design of the study restricts the ability to establish causality between perinatal factors and infant growth. Second, the reliance on maternal self-reports of feeding practices may introduce bias. Third, potential perinatal factors that could influence postnatal growth, such as the quantity and quality of complementary food and family social and economic circumstances, may not have been documented. Further cohort studies are needed to validate the impact and duration of gestational age at birth and early feeding practices on the short-term and long-term health of children.

## Conclusions

In conclusion, this study revealed the link between early-term birth and an elevated risk of overweight and obesity during infancy, as well as the correlation between exclusive breastfeeding and a reduced risk of moderate and severe acute malnutrition in infants under 6 months of age. These findings highlight the significance of avoiding nonmedically indicated deliveries before 39 weeks and further advocating for exclusive breastfeeding before 6 months of age.

## Data Availability

No datasets were generated or analysed during the current study.

## Abbreviations

CI: Confidence Interval
OR: Odds Ratio
GA: Gestational Age
WHO: World Health Organization
LAZ: Length-for-Age Z-Score
WAZ: Weight-for-Age Z-Score
WLZ: Weight-for-Length Z-Score
MSS: Moderate and Severe Stunting
MSAM: Moderate and Severe Acute Malnutrition
GDM: Gestational Diabetes Mellitus
